# Comparing Interrater reliability between eye examination and eye self-examination ^1^

**DOI:** 10.1590/1518-8345.1232.2966

**Published:** 2017-10-19

**Authors:** Maria Alzete de Lima, Lorita Marlena Freitag Pagliuca, Jennara Cândido do Nascimento, Joselany Áfio Caetano

**Affiliations:** 2 PhD, Adjunct Professor, Departamento de Enfermagem, Universidade Federal do Rio Grande do Norte, Natal, RN, Brazil.; 3 PhD, Full Professor, Departamento de Enfermagem, Universidade Federa do Ceará, Fortaleza, CE, Brazil.; 4 PhD, Assistant Professor, Centro Universitário Estácio do Ceará, Fortaleza, CE, Brazil.; 5 PhD, Adjunct Professor, Departamento de Enfermagem, Universidade Federa do Ceará, Fortaleza, CE, Brazil.

**Keywords:** Nursing, Eye Health, Self-Examination, Educational Technology, Ophthalmology, Self Care.

## Abstract

**Objective:**

to compare Interrater reliability concerning two eye assessment methods.

**Method:**

quasi-experimental study conducted with 324 college students including eye self-examination and eye assessment performed by the researchers in a public university. Kappa coefficient was used to verify agreement.

**Results:**

reliability coefficients between Interraters ranged from 0.85 to 0.95, with statistical significance at 0.05. The exams to check for near acuity and peripheral vision presented a reasonable kappa >0.2. The remaining coefficients were higher, ranging from very to totally reliable.

**Conclusion:**

comparatively, the results of both methods were similar. The virtual manual on eye self-examination can be used to screen for eye conditions.

## Introduction

Eye disorders are usually related to chronic health conditions and present multi-varied etiologies. The onset of eye conditions is insidious so that people often do not notice symptoms and, for this reason, their search for specialized care services is delayed, resulting in complications or irreversible visual loss^1^. The incidence of blindness may affect 2 million people every year. Estimates show that if sufficient resources are not invested in prevention, the number of cases may double in the next 10 years^2^. 

The share of the population composed of individuals with moderate or subclinical eye disorders is unknown. Figuratively speaking, it is assumed that these individuals represent only the tip of the iceberg, that is, the population with eye disorders is much larger, so we cannot accurately describe the real magnitude of eye disorders.

This alarming situation is aggravated by increased life expectancy and increased population, a scarcity of specialized services, difficult access to eye care for the population, economic problems and/or a lack of educational programs promoting the adoption of preventive measures^3^. 

Given the evidence that people respond better and are more prone to make decisions when educational strategies using diversified material that favor inclusion, interactivity and accessibility are used^4^ ^-^ ^5^, we sought to adapt eye self-exams to virtual media and validate them with experts and test them with nursing students. The idea is to enable people to identify eye problems in order to seek specialized care at an earlier point in time. For this strategy to be successful, however, assessment of Interrater reliability is necessary to determine whether the results of self-exams are consistent with examinations performed by trained staff. 

Eye self-examination enables people to understand their eyes and identify changes that may occur in visual acuity, external eye structures, visual field and eye movement. Hence, health workers should encourage the practice. The relevance of this study is that it makes technology providing eye assessment available and encourages the search for specialized care when warning signs are detected. The objective was to compare Interrater reliability of two eye assessment methods. The hypothesis of interest is that eye examinations performed by specialized staff, considered to be the gold standard, obtain results that are compatible with self-exams when using a virtual manual.

## Method

This quasi-experimental study focuses on the assessment of Interrater reliability concerning an eye examination technique performed by a trained researcher. The participants performed an eye self-examination using a virtual manual on eye self-examination. 

Reliability is the extent to which repeated measurements, of a stable phenomenon, performed by different people and/or instruments at different times and places, present similar results. Interrater reliability assesses level of agreement among raters or the consistency of the performance of two or more raters in recording the same answers at the same time^6^. This property was verified in this study using the results of eye assessments performed by the researcher and team. College students using a virtual manual addressing eye self-examination performed the eye self-examination. 

The study was conducted from January to May 2014 in a public university with a population of 2,060 students. This institution was chosen because of the availability of computers, Internet access at the institution and the ease and access the primary author had to conduct data collection.

A convenience sample was used and inclusion criteria were: being in a physical condition to perform the eye self-examination and mastering basic informatics. Those with diagnosed eye conditions or enrolled in programs from the health field were excluded. 

Sampling calculation was carried out considering 95% confidence level, estimated proportion of success of 50%, and 5% level of precision, resulting in a sample size of 324 observations, according to the formula for finite populations:



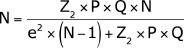



Considering the risk of communication among participants regarding the exam to be performed, and aiming to avoid data collection bias, students from different classes, programs and shifts were invited in alternated weeks. Data were collected from seven programs, which were stratified according to the number of students in order to ensure representativeness. 

**Table 1:** Stratified sample according to program and working hours

**Program**	**Total students**	Time*	**Sample**
**Biology**	**427**	**A/N**	**88**
**Mathematics**	**227**	**A/N**	**24**
**Information system**	**273**	**M/A**	**34**
**Business administration**	**451**	**M/A**	**98**
**History**	**329**	**A/N**	**51**
**Letters**	**179**	**A/N**	**16**
**Pedagogy**	**174**	**A/N**	**13**

Source: Developed by the author *M - Morning; A- Afternoon; N-Night.

The manual provided instructions on how to perform an eye self-exam providing simple information that enabled the identification of eye problems. It described the technique to assess visual acuity (near and distance), external eye structures, visual field (peripheral and central vision), and eye movement. These stages intended to identify potential changes such as diminished visual acuity, lesions, loss of visual field, strabismus, diplopia, and redness, among others^7^. The students assessed the computer in the laboratory of clinical practices and the following material for the self-exam were placed on a table: Snellen scale, 5m measurement tape, adhesive tape, eye occlude, pen and paper, mirror, cotton tip, reading material, and alcohol gel.

The team that collected data was composed of two nurses and 13 students. This team performed the eye assessment after the students performed self-examinations, and the results were recorded on a specific form. This team was trained with classes that focused on the semiotics of the visual apparatus, eye exam in loco, and how to complete the form, totaling 20 hours. The students presenting results that indicated eye problems were referred to a Family Health Strategy unit linked to the university. 

Data were entered into Microsoft Excel using a triple typing technique, which after verification of the consistency of data, were transferred to the Statistical Package for Social Science (SPSS), version 20.0. In order to observe the results of the two methods, each individual was his/her own control, with matched analysis. The kappa coefficient was used to assess Interrater reliability and Landis and Koch classification was used in the interpretation, which determines 1 for κ < 0.00 (no reliability); 2 for κ between 0.00 and 0.20 (weak reliability); 3 for κ between 0.21 and 0.40 (reasonable); 4 for κ between 0.41 and 0.60 (regular); 5 for κ between 0.61 and 0.80 (good); 6 for κ between 0.81 and 0.99 (excellent); 7 for κ = 1.00 (perfect). Level of significance of 0.05 was adopted. 

The results were organized into a contingency table, which shows the results of the exams performed by the participants and the team. Position A indicates a true-positive result, that is, both evaluators verified the result was normal, indicating the student was accurate in his/her judgment; position B refers to false-positive results and indicates the student considered the exam to be normal when in reality there was an eye problem; position C indicates the student considered there to be an eye condition when in reality the result was normal; and position D, true-negative, indicates there was an eye condition and the judgment of both evaluators agreed. 

The study was approved by the Institutional Review Board under protocol 508,069. The students received clarification about the study and signed free and informed consent forms.

## Results

Of the 324 students, 193 (59.6%) were male, 294 (91.0%) were single, and 279 (86.1%) were considered young, aged 21 years old on average, with a standard deviation of 3.3 years. The coefficient of variation was low (15.5%), indicating the distribution of this variable was homogeneous.

Table 2 shows 34 false-positive and 51 false-negative results in the assessment of distance visual acuity. Even though Kappa >0.6 and categorization showed a high level of agreement, proportionally, even with the use of the manual, this exam presented a higher likelihood of presenting an interpretation error compared to a near acuity vision assessment. 

**Table 2:** Test of agreement between self-examination and eye exam performed by the researcher according to Kappa (n = 324). Picos, PI, Brazil, 2014

Exams			Possible results				Kappa Index	*P*-value	Landis and Koch category	
			Normal and normal (A)		Normal and altered (B)	Altered and normal (C)	Altered and altered (D)			
Visual acuity										
	Right eye									
		Distance		256	13	21	34	0.605	<0.001	5
		Near		296	7	15	6	0.318	<0.001	3
	Left eye									
		Distance		232	21	30	41	0.518	<0.001	4
		Near		297	7	15	5	0.279	<0.001	3
Eye structures										
	Right eye									
		Eyelid		324	0	0	0	-	-	-
		Eyelashes		322	0	2	0	-	-	-
		Conjunctiva		317	1	3	3	0.594	<0.001	4
		Sclera		304	5	8	7	0.498	<0.001	4
		Cornea		323	1	0	0	-	-	-
		Pupil		312	2	6	4	0.488	<0.001	4
		Iris		323	0	1	0	-	-	-
	Left eye									
		Eyelid		321	0	0	3	1.000	<0.001	7
		Eyelashes		324	0	0	0	-	-	-
		Conjunctiva		317	1	3	3	0.594	<0.001	4
		Sclera		307	5	7	5	0.436	<0.001	4
		Cornea		324	0	0	0	-	-	-
		Pupil		311	4	3	6	0.620	<0.001	5
		Iris		322	0	2	0	-	-	-
Eye movement										
	Right eye			313	0	6	5	0.617	<0.001	5
	Left eye			312	1	7	4	0.489	<0.001	4
Visual field										
	Right eye									
		Central vision		302	7	6	9	0.561	<0.001	4
		Peripheral vision		269	2	41	12	0.311	<0.001	3
	Left eye									
		Central vision		303	4	8	9	0.581	<0.001	4
		Peripheral vision		259	6	45	14	0.289	<0.001	3

In regard to examination of the external eye structures, Interrater agreement did not enable randomization of data and consequent statistical testing, with exception of the exam for conjunctiva, sclera and pupil, in which level of agreement ranged between regular and good.

In regard to eye structures and eye movement, the results were practically the same; that is, the number of false-positive and negative results was not significant considering the high level of agreement between results.

The results concerning assessment of the visual field show reasonable to regular level of agreement. Still, more than 273 students presented the same results as those found by the expert team, including true-positive and negative results.

The manual shows that the distance visual acuity exams and those of the visual field for peripheral eyesight need to be reviewed, as a high proportion of disagreement was found among the tests’ results. Nonetheless, the use of the virtual educational manual enabled individuals to perform the eye self-exam, as well as the examination performed by the health team.

## Discussion

Self-examination is a self-care strategy that has proven benefits for the survival of many individuals using internet-based self-management^8^. A simple assessment of eye acuity evidences the functional integrity of the eye system in its entire complexity and is considered an important screening element for varied eye disorders, a reference factor to monitor the efficacy of treatments^9^.

Poor eyesight caused by refractive errors is recognized worldwide as an important cause of avoidable visual impairment. Myopia is one of the most common disorders in the world, being more prevalent among individuals with higher educational level(9), hence, its early identification is essential. Assessment of eye structures also enables the prevention of severe eye complications^10^. This detection can also reveal the incidence of plasma cell dyscrasia, for instance, which can be an etiological factor in senile cataract and glaucoma^11^.

A presymptomatic eye assessment, which identifies age-related cataracts, for example, can promote preventive measures, as the eye is easily accessible for the topical application of medication. Its potential is translated as pharmacologically practical prevention or even the treatment of cataracts^12^.

Hence, easy and reliable ophthalmic screening should be implemented in schools and institutions and be part of government actions. It’s implementation should be widely disseminated in developing and developed countries, considering that the late diagnosis of eye conditions may impact quality of life and result in costs in the health field^13^. 

The virtual manual presented positive effects regarding the correct way to perform a self-exam and can be considered to have the potential to expand clinical results, as it is in agreement with new conceptions of health care management^14^.

## Conclusion

In this study, eye assessment performed by trained people was compared to college students’ self-examinations and Interrater reliability was attested as Kappa index concerning level of agreement ranged from regular to total reliable. 

There are some limitations, however, such as the time between the development of the manual and it’s assessment: the manual’s development did not follow the rapidly expanding availability of technological innovation and there is a lack of assessment indexes enabling the measurement, through a scale, of the manual’s level of reliability. Nonetheless, the study’s purpose was achieved, as the manual is shown to be reliable in the use of technology and knowledge of healthcare, essential to disseminating eye self-exam and encouraging adherence to it, anchoring the individuals’ cognitive structure, that is, providing efficient anchoring. 

This study shows the use of the Internet and educational media is a reality worldwide and is booming in the fields of health, but investment in the nursing field is needed. 
